# Elevated carbon dioxide enhances the growth and reduces the antifungal susceptibility of *Histoplasma capsulatum*

**DOI:** 10.1128/spectrum.03106-24

**Published:** 2025-05-30

**Authors:** Qian Shen, Kelsey Steinmetz

**Affiliations:** 1 Department of Biology, Rhodes College 5414, Memphis, Tennessee, USA; 2 Biochemistry and Molecular Biology Program, Rhodes College 5414, Memphis, Tennessee, USA; Universidade de Sao Paulo, Sao Paulo, Brazil

**Keywords:** carbon dioxide, *Histoplasma*, antifungal susceptibility, growth

## Abstract

*Histoplasma capsulatum* is a thermally dimorphic fungal pathogen. It causes approximately 500,000 infections annually in the United States. *Histoplasma* is present as avirulent mycelia in the soil and transforms into pathogenic yeasts at the human body temperature upon inhalation. This elevated temperature triggers the expression of many virulence factors that enable *Histoplasma* yeasts to survive and proliferate within immune cells (i.e., macrophages) in the human lungs. In addition to elevated temperature, *Histoplasma* yeasts also experience other environmental changes within the mammalian host, such as elevated carbon dioxide (CO_2_) (ambient air vs host tissues) during infection. However, the impact of elevated CO_2_ on *Histoplasma* yeasts remains completely unknown. In this study, our results showed that elevated CO_2_ enhanced *Histoplasma*’s growth, particularly increasing its ability to utilize certain amino acids (e.g., alanine) as the sole carbon source. We also found that elevated CO_2_ reduced *Histoplasma*’s susceptibility to antifungals *in vitro*. *Histoplasma*’s enhanced growth and reduced antifungal susceptibility under elevated CO_2_ were not pH-dependent. Our findings suggest that the elevated CO_2_ within mammalian hosts could potentially enhance *Histoplasma*’s virulence. Future antifungal susceptibility tests for *Histoplasma* should be performed at 5% CO_2_ for clinically relevant results.

**IMPORTANCE:**

The fungal pathogen *Histoplasma capsulatum* lives in the soil. *Histoplasma* spores can be inhaled and cause respiratory infections. The human body is vastly different from the soil. One of the major differences is the carbon dioxide (CO_2_) concentration (0.04% in the ambient air vs 5% or above in the human body). Therefore, it is important to understand the impact of elevated CO_2_ on *Histoplasma*. We found that elevated CO_2_ promotes *Histoplasma*’s growth, suggesting that elevated CO_2_ could potentially enhance *Histoplasma*’s virulence during infection. Our results showed that elevated CO_2_ reduces *Histoplasma*’s antifungal susceptibility, suggesting that antifungal susceptibility tests for *Histoplasma* should be performed at elevated CO_2_ for clinically relevant results.

## INTRODUCTION

*Histoplasma capsulatum* is a dimorphic fungal pathogen that causes respiratory infection in both immunocompetent and immunocompromised individuals (1). Among individuals who are hospitalized with histoplasmosis, the mortality rates generally range from 5% to 7% (U.S. Centers for Disease Control and Prevention). Immunocompromised individuals (e.g., HIV patients and organ transplant recipients) have the most significant risk of developing a life-threatening systemic infection (2–6). *Histoplasma* is endemic to many regions of the world, including regions of North, Central, and South America (2, 7) as well as regions of Asia and Africa (8–10). *H. capsulatum* can be classified into distinct clades that correlate with their region of geographic isolation (11, 12). *H. capsulatum* can also be classified into two chemotypes based on its cell wall composition. Isolates with or without the polysaccharide α-(1, 3)-glucan in their cell wall are designated chemotype II and chemotype I, respectively (13). *H. capsulatum* G186A and G217B are the representative strains for chemotype II and chemotype I, respectively.

*Histoplasma* is found in the soil as mycelia. It produces conidia, which can be inhaled by the mammalian host. The elevated temperatures within the mammalian host prompt the conidia to differentiate into pathogenic yeasts (14–17). Unlike other fungal pathogens (e.g., *Candida albicans* and *Aspergillus fumigatus*) that are readily controlled by innate immunity, *Histoplasma* yeasts proliferate within the phagosomal compartment of alveolar macrophages (5, 18). This phagosomal environment differs greatly from the soil. The temperature of the mammalian host (e.g., 37°C) is higher than that in the soil. Furthermore, the phagosomal compartment within alveolar macrophages is a nutrient-depleted environment (19–21). The temperature-induced morphological change upregulates certain genes that help *Histoplasma* combat nutritional limitations within the macrophage phagosome (22). For example, *Histoplasma* yeasts upregulate *SID1* (22–24)*, ZRT2* (22, 25), and *CTR3* (22, 26) to acquire sufficient iron, zinc, and copper, respectively, during infection.

In addition to elevated temperature and limited nutrition, *Histoplasma* yeasts also experience a dramatic increase in CO_2_ levels within the mammalian host. CO_2_ plays an important metabolic role and serves as a crucial signaling molecule in fungi. For instance, the bicarbonate ions generated from CO_2_ by carbonic anhydrases are key substrates for the carboxylation of enzymes (e.g., acetyl-CoA carboxylase), central to many metabolic processes, including fatty acid biosynthesis (27). CO_2_ also impacts various other cellular processes in fungi, including nutrient acquisition and mating (28–30). More importantly, CO_2_ behaves as a signaling molecule to enhance the virulence of several human fungal pathogens (28). For instance, CO_2_ activates adenylyl cyclase to enhance the virulence of *C. albicans* by promoting the morphological transition from yeast to hyphae (30–32). CO_2_ stimulates capsule production, a major virulence factor, in the fungal pathogen, *Cryptococcus neoformans* (33, 34). In addition to virulence, elevated CO_2_ alters the susceptibility of *Cryptococcus* to antifungal drugs such as azoles and flucytosine (35–37). However, the influence of elevated CO_2_ has never been studied in the primary fungal pathogen *H. capsulatum*. Therefore, this study aims to investigate how elevated CO_2_ impacts *Histoplasma*’s growth and its antifungal susceptibility.

## RESULTS

### Elevated CO_2_ enhances *Histoplasma*’s growth

**Fig 1:**
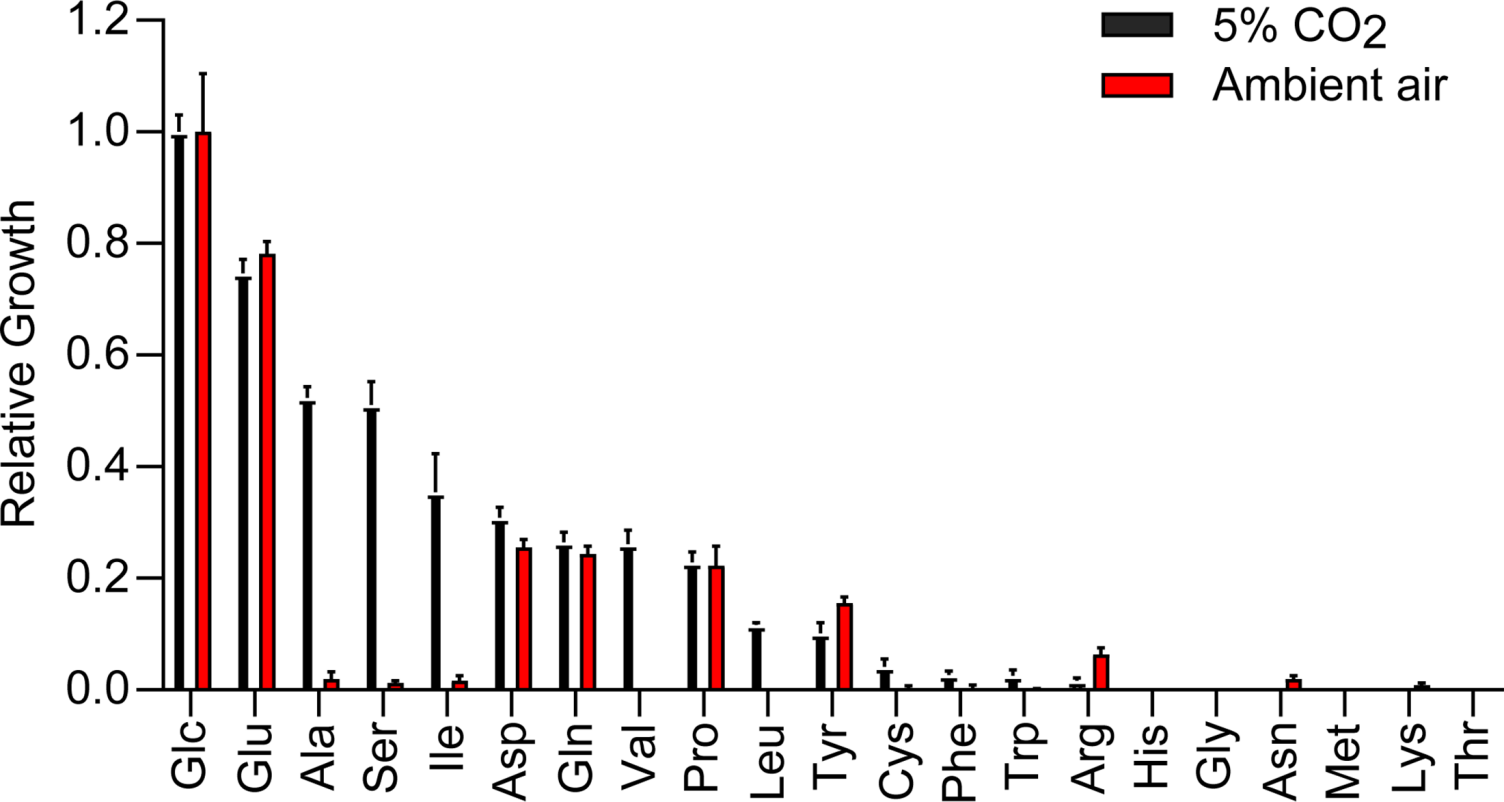
The growth of *Histoplasma* yeasts in individual amino acid as the sole carbon source. *Histoplasma* yeasts were inoculated into 3M medium at 2 × 10^6^ yeasts/mL containing each individual amino acid as the sole carbon source. Glucose was used as the positive control. Glucose or each individual amino acid provides 250 mM of carbon source except for cysteine (1.5 mM) and tyrosine (1.25 mM). Yeasts were incubated at 37°C under 5% CO_2_ or ambient air (0.04% CO_2_). Yeast growth was measured by determination of the optical density at 595 nm (OD_595_) after 7 days of incubation and normalized to the growth in glucose. Data represent average relative growth levels ± standard deviations of results from biological replicates (*n* = 3).

**Fig 2:**
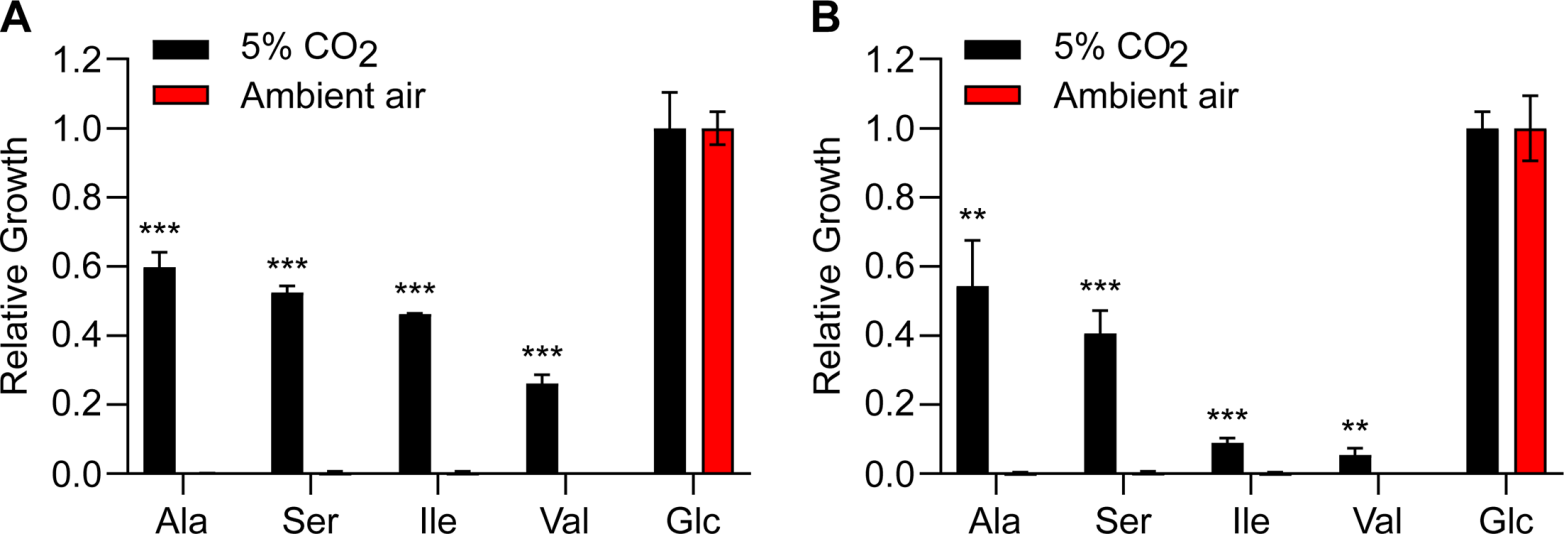
Elevated CO_2_ enhances the growth of *Histoplasma* in amino acids. The yeast growth of two distinct lineages of *Histoplasma* ((**A**), G217B; (**B**), G186A) was determined in alanine (Ala), serine (Ser), isoleucine (Ile), or valine (Val) as the sole carbon source. *Histoplasma* yeasts were inoculated into 3M medium at 2 × 10^6^ yeasts/mL containing each individual amino acid as the sole carbon source. Glucose was used as the positive control. Each individual amino acid or glucose provides 250 mM of carbon source. Yeasts were incubated at 37°C under 5% CO_2_ or ambient air (0.04% CO_2_). Yeast growth was measured by determination of the optical density at 595 nm (OD_595_) after 7 days of incubation and normalized to the growth in glucose. Data represent average relative growth levels ± standard deviations of results from biological replicates (*n* = 3). Asterisks indicate significant differences (**, *P* < 0.01; ***, *P* < 0.001) between 5% CO_2_ and ambient air as determined by two-tailed Student’s *t*-test.

*Histoplasma* yeasts were grown in 3M medium containing individual amino acids as the sole carbon source under either ambient air or 5% CO_2_. The growth of *Histoplasma* yeasts in alanine, serine, isoleucine, and valine was greatly enhanced under 5% CO_2_ compared to that under ambient air (Fig. 1). Similar results were also observed in the Panama lineage G186A yeasts (Fig. 2). *Histoplasma* yeasts can grow in 3M medium with alanine, serine, isoleucine, and valine as the sole nitrogen source under ambient air (Fig. S1), indicating that *Histoplasma* yeasts can uptake these amino acids and utilize the amino group as the sole nitrogen source. Therefore, the lack of growth of *Histoplasma* yeasts in alanine, serine, isoleucine, and valine under ambient air is largely due to their inability to metabolize the carbon skeleton of these amino acids.

**Fig 3:**
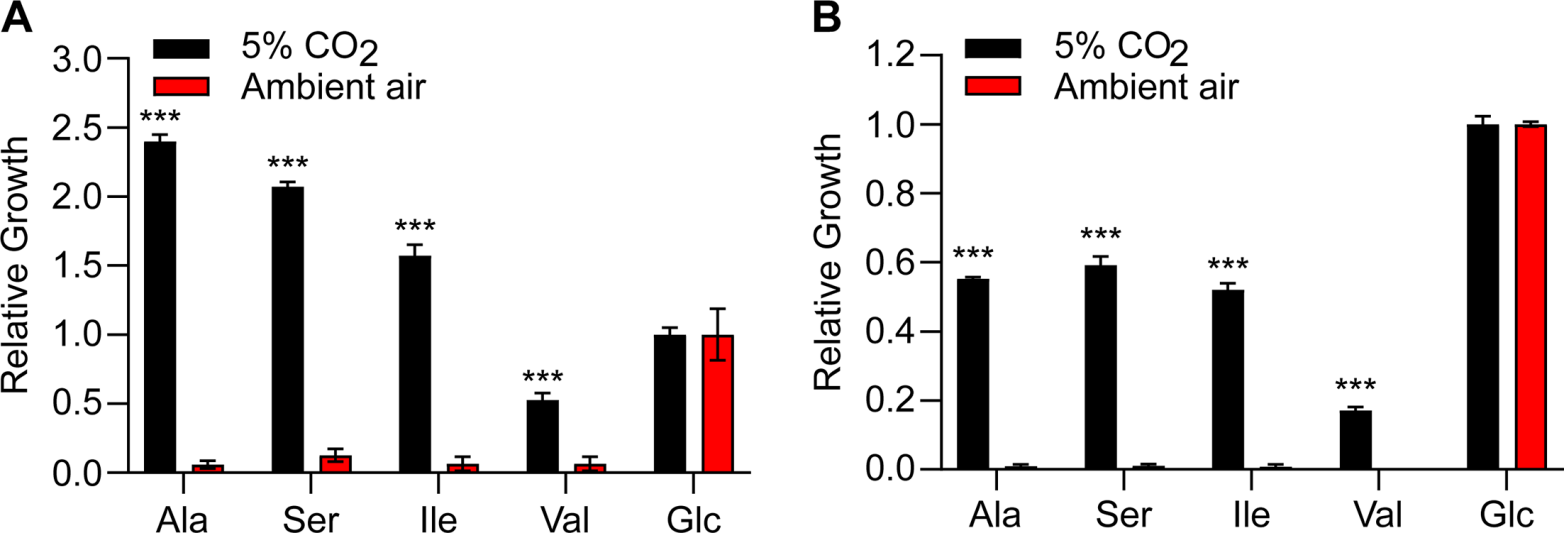
Enhanced *Histoplasma* growth in amino acids under elevated CO_2_ is not pH-dependent. The yeast growth of *Histoplasma* in alanine (Ala), serine (Ser), isoleucine (Ile), or valine (Val) as the sole carbon source was determined under pH 5.0 (**A**) or pH 7.0 (**B**). *Histoplasma* yeasts were inoculated into 3M medium at 2 × 10^6^ yeasts/mL containing each individual amino acid as the sole carbon source. Glucose was used as the positive control. Each individual amino acid or glucose provides 250 mM of carbon source. Yeasts were incubated at 37°C under 5% CO_2_ or ambient air (0.04% CO_2_). The 3M medium with pH 5.0 or 7.0 was buffered by 20 mM MES or 20 mM HEPES, respectively. Yeast growth was measured by determination of the optical density at 595 nm (OD_595_) after 7 days of incubation and normalized to the growth in glucose. Data represent average relative growth levels ± standard deviations of results from biological replicates (*n* = 3). Asterisks indicate significant differences (***, *P* < 0.001) between 5% CO_2_ and ambient air as determined by two-tailed Student’s *t*-test.

In an aqueous solution, elevated CO_2_ increases the concentration of carbonic acid, which dissociates into bicarbonate ions and protons, resulting in acidification of the solution. Therefore, we tested whether the enhanced growth of *Histoplasma* yeasts in alanine, serine, isoleucine, and valine under 5% CO_2_ is pH-dependent. *Histoplasma* yeasts showed enhanced growth in these amino acids under 5% CO_2_ compared to ambient air at both pH 5 and 7 (Fig. 3), with enhanced growth overall at pH 5. This demonstrates that the growth advantage in alanine, serine, isoleucine, and valine does not result from CO_2_-induced pH changes.

**Fig 4:**
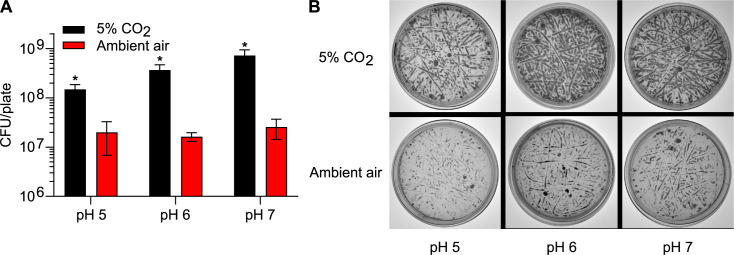
Elevated CO_2_ enhances the growth of *Histoplasma* on the solid medium. *Histoplasma* yeasts (5 × 10^6^ yeasts) were inoculated onto the HMM agar with pH 5, 6, and 7, respectively, and incubated at 37°C under 5% CO_2_ or ambient air (0.04% CO_2_). *Histoplasma* yeast growth (dark area) after 72 h on the HMM agar was measured by determining the total viable yeast count on each agar plate. Data represent average total viable yeast count ±standard deviations of results from biological replicates (*n* = 3). Asterisks indicate significant differences (*, *P* < 0.05) between 5% CO_2_ and ambient air as determined by Welch’s *t*-test (**A**). Representative images of *Histoplasma* yeast growth after 72 h on the HMM agar with pH 5, 6, and 7, respectively, under 5% CO_2_ or ambient air were shown (**B**).

*Histoplasma* yeasts also showed enhanced growth on solid rich medium (*Histoplasma*-macrophage medium [HMM] agar) under 5% CO_2_ (Fig. 4). This enhanced growth was also observed in the Panama lineage G186A yeasts as well, but to a lesser extent (Fig. S2). This result is qualitative because there is no protocol available to accurately quantify the viable G186A yeast numbers from the HMM agar due to G186A’s tendency to form large clumps. Even though HMM has ample glucose, the enhanced growth of *Histoplasma* yeasts under 5% CO_2_ suggests that *Histoplasma* yeasts prefer utilizing certain amino acids (e.g., alanine or serine) for growth *in vitro*. Surprisingly, while enhanced growth under 5% CO_2_ was observed for both *Histoplasma* lineages on solid HMM (Fig. 4 and Fig. S2), the growth advantage under 5% CO_2_ disappeared when the yeasts were grown in liquid HMM (Fig. S3). This is potentially due to the CO_2_ generated from cellular respiration being trapped in the liquid medium, thereby increasing the CO_2_ concentration in the liquid medium despite being under ambient air.

### Elevated CO_2_ reduces *Histoplasma*’s antifungal susceptibility

**Fig 5:**
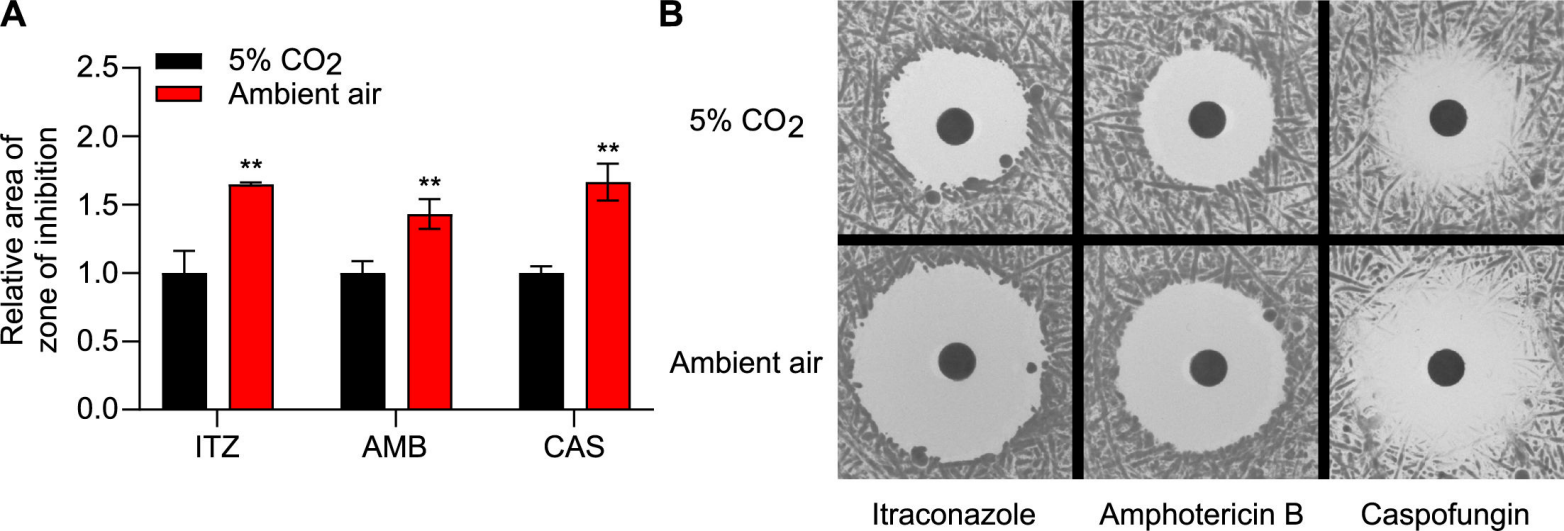
Elevated CO_2_ reduces susceptibility to antifungals in *Histoplasma*. The antifungal susceptibility of *Histoplasma* under 5% CO_2_ or ambient air (0.04% CO_2_) was determined using a disk diffusion assay. Suspensions of *Histoplasma* yeasts (2 × 10^7^ cells) were spread onto the HMM agar (pH 6). Disks containing ITZ (itraconazole, 32 µg/ml), AMB (amphotericin B, 150 µg/mL), and CAS (caspofungin, 6.4 mg/mL) were placed on top of the spread cells. The cells were incubated at 37°C under 5% CO_2_ or ambient air. The area of the zone of inhibition was measured after 5 days and normalized to the area of the zone of inhibition under 5% CO_2_. Larger zone of inhibition indicates greater antifungal susceptibility. Data represent average relative area of zone of inhibition ±standard deviations of results from biological replicates (*n* = 3). Asterisks indicate significant differences (**, *P* < 0.01) between 5% CO_2_ and ambient air as determined by two-tailed Student’s *t*-test (**A**). Representative images of *Histoplasma*’s susceptibility to itraconazole, amphotericin B, or caspofungin under 5% CO_2_ or ambient air (0.04% CO_2_) were shown (**B**).

The effect of CO_2_ levels on the antifungal susceptibility of *Histoplasma* was determined by a disk diffusion assay using itraconazole (azole), amphotericin B (polyene), and caspofungin (echinocandin). Under 5% CO_2_, the susceptibility of *Histoplasma* yeasts to all three antifungals decreased compared to that under ambient air (Fig. 5). Our previous work suggests that *Histoplasma* yeasts likely use amino acids as the major carbon source during infection (21). Therefore, we also tested whether the reduced susceptibility of *Histoplasma* yeasts under 5% CO_2_ still holds true in a medium with amino acids as the only carbon source. We found that elevated CO_2_ reduces *Histoplasma*’s susceptibility to itraconazole and caspofungin, but not amphotericin B in the amino acid medium (Fig. S4). The Panama lineage G186A yeasts showed reduced susceptibility to itraconazole, but not amphotericin B under 5% CO_2_ (Fig. S5). The effect of CO_2_ on the susceptibility of G186A yeasts to caspofungin was not reported as these yeasts showed high resistance to caspofungin in the disk diffusion assay.

**Fig 6:**
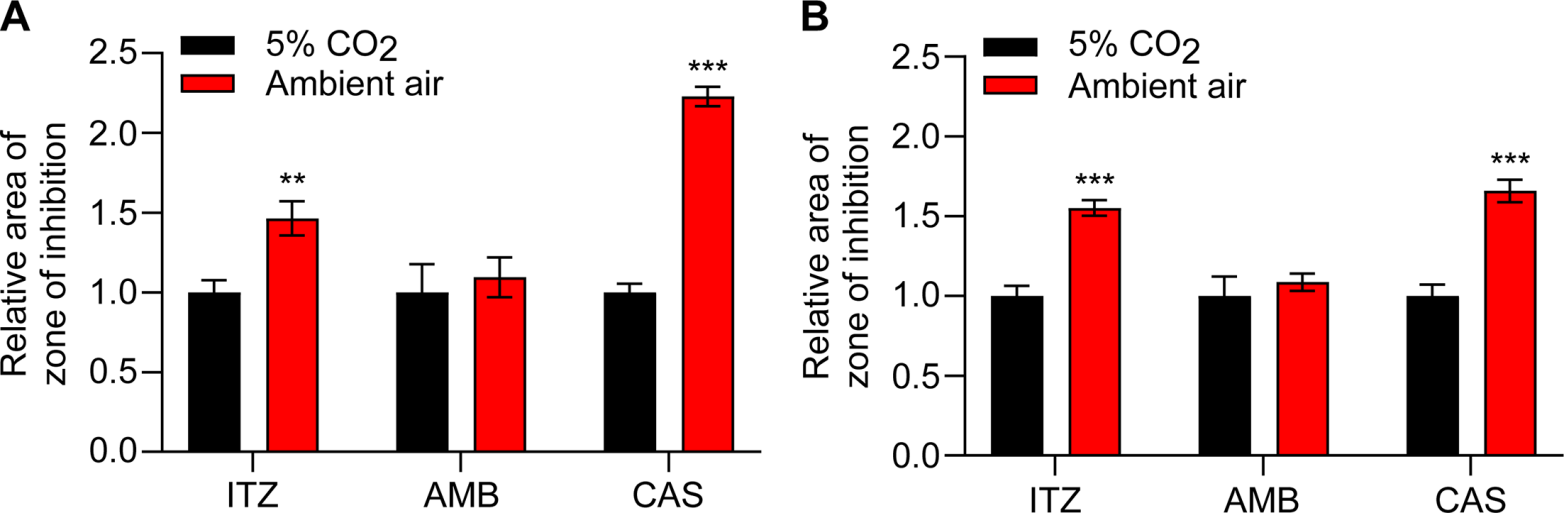
*Histoplasma*’s reduced susceptibility to itraconazole and caspofungin under elevated CO_2_ is not pH-dependent. The antifungal susceptibility of *Histoplasma* under 5% CO_2_ or ambient air (0.04% CO_2_) was determined at pH 5.0 (**A**) or pH 7.0 (**B**) using a disk diffusion assay. The solid HMM with pH 5.0 or 7.0 was buffered by 20 mM MES or 20 mM HEPES, respectively. Suspensions of *Histoplasma* yeasts (2 × 10^7^ cells) were spread onto the solid HMM. Disks containing ITZ (itraconazole, 32 µg/mL), AMB (amphotericin B, 150 µg/mL), and CAS (caspofungin, 6.4 mg/mL) were placed on top of the spread cells. The cells were incubated at 37°C under 5% CO_2_ or ambient air. The area of the zone of inhibition was measured after 5 days and normalized to the area of the zone of inhibition under 5% CO_2_. Larger zone of inhibition indicates greater antifungal susceptibility. Data represent average relative area of zone of inhibition ±standard deviations of results from biological replicates (*n* = 3). Asterisks indicate significant differences (**, *P* < 0.01; ***, *P* < 0.001) between 5% CO_2_ and ambient air as determined by two-tailed Student’s *t*-test.

The disk diffusion assay was repeated at both pH 5 and 7 to determine if this reduced susceptibility under 5% CO_2_ is pH-dependent. Under both pH 5 and 7, *Histoplasma* yeasts consistently showed reduced susceptibility to itraconazole and caspofungin under 5% CO_2_ (Fig. 6). However, the reduced susceptibility of *Histoplasma* yeasts to amphotericin B under 5% CO_2_ appeared to be pH-dependent (Fig. 6). Interestingly, the decreased antifungal susceptibility under 5% CO_2_ was not observed for *Histoplasma* yeasts grown in liquid HMM (Fig. S6). This could be due to the trapped CO_2_ from cellular respiration under ambient air, which is consistent with the absence of the enhanced growth phenotype under 5% CO_2_ in liquid HMM (Fig. S3).

## DISCUSSION

Microbial pathogens must adapt to the mammalian host environment to successfully establish infections. The habitat of *Histoplasma* in the soil is vastly different from the host environment during infection. Differences such as temperature, nutrient availability, and level of CO_2_ can significantly impact the physiology and potentially the virulence of *Histoplasma*. In this study, we examined the impact of elevated CO_2_ similar to that of the host environment on *Histoplasma* yeasts. Our data demonstrated that elevated CO_2_ enhances *Histoplasma*’s growth. A previous study reported similar findings, showing that elevated CO_2_ levels promote the growth of *Histoplasma* yeasts in liquid media, possibly as a result of pH changes induced by the shift of CO_2_ concentration (38). In *Coccidioides immitis*, elevated CO_2_ is required for the development and the maintenance of spherules (39, 40).

The enhanced growth under elevated CO_2_ was observed in medium with certain amino acids (i.e., alanine, serine, isoleucine, and valine) as the sole carbon source (Fig. 1 and 2). Interestingly, the catabolism of alanine, serine, isoleucine, and valine converges on the common metabolite pyruvate, which can be converted into oxaloacetate for subsequent gluconeogenesis, a process which is essential for *Histoplasma*’s growth on amino acids as the sole carbon source. The reaction catalyzed by pyruvate carboxylase requires the substrate bicarbonate, which is the product of dissolved CO_2_. This leads to a model that the lower CO_2_ concentration in ambient air does not provide sufficient bicarbonate for this carboxylation reaction to produce oxaloacetate, resulting in impaired gluconeogenesis when grown in media with amino acids as the only carbon source. This is consistent with our results that amino acids such as glutamate and aspartate that can produce oxaloacetate without pyruvate carboxylase support *Histoplasma*’s growth under ambient air (Fig. 1). The low CO_2_ concentration in ambient air does not appear to affect other carboxylation processes that are involved in the biosynthesis of fatty acids, arginine, purines, and pyrimidines (27), as *Histoplasma* yeasts can grow in glucose as the sole carbon source under ambient air (Fig. 1).

In this study, we also found that elevated CO_2_ reduced *Histoplasma*’s antifungal susceptibility (Fig. 5). This reduced susceptibility under elevated CO_2_ to amphotericin B was pH-dependent, whereas the reduced susceptibility to itraconazole and caspofungin was consistent under all pHs and all types of media tested (Fig. 5 and 6). The reduced susceptibility to caspofungin (Fig. 5) suggests that elevated CO_2_ might have an impact on *Histoplasma*’s cell wall structure. In *C. albicans*, the cellular components (Nce103, Rca1, and Sch9) that are responsible for CO_2_ sensing modulate β-1,3-glucan exposure under elevated CO_2_ (41). In contrast to *Histoplasma*, *C. neoformans* showed increased antifungal susceptibility under elevated CO_2_ (35–37). This increased susceptibility was consistent among different types of antifungals, including fluconazole, itraconazole, myriocin, and flucytosine (35–37), suggesting increased uptake of antifungals under elevated CO_2_. Consistent with this, the cytosine permease gene (*FCY2*) in *C. neoformans*, encoding a flucytosine transporter, was upregulated under elevated CO_2_ (36). The opposite phenotype observed in our study suggests that *Histoplasma* has a unique cellular response to elevated CO_2_, which is consistent with the fact that *Histoplasma* (Ascomycota) is evolutionarily quite divergent from *C. neoformans* (Basidiomycota).

The CO_2_-induced enhanced growth and reduced antifungal susceptibility were not observed when *Histoplasma* yeasts were grown in the liquid medium (Fig. S3 and S6). *Histoplasma* yeasts grown in the 96-well plate tend to settle at the bottom of the well, thus experiencing low oxygen concentration due to the lack of constant aeration. Oxygen concentration can affect the growth and the antifungal susceptibility of other fungal pathogens (42–44). It is possible that the cellular response to reduced oxygen concentration (e.g., hypoxia) interferes with the cellular response to elevated CO_2_. In our future studies, we will remove the gene *SRB1*, encoding a protein involved in adaptation to low oxygen levels, in *Histoplasma* (45) and determine whether this mutant can restore the CO_2_-induced enhanced growth and reduced antifungal susceptibility in the liquid medium.

Our previous work indicates that amino acids are the major carbon source that can support *Histoplasma*’s growth within macrophages during infection (21). Therefore, the enhanced growth in certain amino acids *in vitro* under physiologically relevant CO_2_ might suggest a novel mechanism to promote *Histoplasma* yeast intracellular growth in an amino acid-sufficient environment during infection. Furthermore, the reduced antifungal susceptibility of *Histoplasma* under elevated CO_2_ *in vitro* suggests that antifungal testing under conditions that mimic the host environment provides more accurate information about the drug susceptibility profiles of *Histoplasma* clinical isolates. The underlying mechanisms by which elevated CO_2_ enhances *Histoplasma*’s growth and resistance to antifungals are unknown. Since these phenotypes are unlikely caused by the change of pH under elevated CO_2_, future studies should focus on how *Histoplasma* yeasts sense elevated CO_2_ (e.g., direct CO_2_, bicarbonate, or both), and the network of genes and proteins that mediate the response.

## MATERIALS AND METHODS

### *Histoplasma* strains and growth

The *Histoplasma* strains used in this study were the North American clade 2 clinical isolate G217B and the Panama strain G186A. For general maintenance of strains, *Histoplasma* yeasts were grown in HMM (46). For growth on solid medium, HMM was solidified with 0.6% agarose and supplemented with 25 µM FeSO_4_. HMM was adjusted to pH 6 unless otherwise noted. For growth curve determination, yeasts were inoculated at 2 × 10^6^ yeasts/mL in 96-well microtiter plates and incubated at 37°C under ambient air or 5% CO_2_ for 5 days with twice-daily agitation (47). Growth of yeasts in liquid culture was quantified by the measurement of culture turbidity (optical density at 595 nm [OD_595_]) every 24 h. The experiments were performed using the North American clade 2 clinical isolate G217B unless otherwise noted.

For growth in amino acid as the sole carbon source, yeasts were grown in 3M medium (46) containing ammonium sulfate as the sole nitrogen source and a limited amount of cysteine (25 µM) as the major sulfur source (21). Each individual amino acid was added into the 3M medium to reach a final carbon concentration of 250 mM unless otherwise noted. Cysteine and tyrosine were used at 1.5 and 1.25 mM, respectively, due to low solubility. Growth in 3M medium with glucose (0.75%, wt/vol) served as a positive control. Yeasts were inoculated at 2 × 10^6^ yeasts/mL in 96-well microtiter plates and incubated at 37°C under ambient air or 5% CO_2_ with twice-daily agitation (47). Growth of yeasts in liquid culture was quantified by the measurement of culture turbidity (OD_595_) after 7 days of incubation. G186A yeasts were treated with 1 M NaOH to disperse clumps before the OD was read. Relative growth in each individual amino acid was calculated by normalizing to the growth in glucose.

For growth in amino acid as the sole nitrogen source, yeasts were grown in 3M medium (46) containing glucose as the sole carbon source and a limited amount of cysteine (25 µM) as the major sulfur source (21). Each individual amino acid was added into the 3M medium to reach a final nitrogen concentration of 7.5 mM unless otherwise noted. Cysteine and tyrosine were used at 1.5 and 1.25 mM, respectively, due to low solubility. Asparagine, histidine, phenylalanine, and methionine were used at 1, 0.25, 0.4, and 0.8 mM, respectively, due to their inhibitory effect at higher concentrations. Growth in 3M medium with 7.5 mM of ammonium sulfate served as a positive control. Yeasts were inoculated at 2 × 10^6^ yeasts/mL in 96-well microtiter plates and incubated at 37°C under ambient air or 5% CO_2_ with twice-daily agitation (47). Growth of yeasts in liquid culture was quantified by the measurement of culture turbidity (OD_595_) after 7 days of incubation. Relative growth in each individual amino acid was calculated by normalizing to the growth in ammonium sulfate.

To quantify growth on solid medium, about 5 × 10^6^ yeasts were spread on petri dishes (60 mm diameter) containing HMM agar and incubated at 37°C under ambient air or 5% CO_2_. After 72 h, the yeasts were harvested by scraping the surface of the HMM agar. Serial dilutions of the yeast cell suspension were plated on solid HMM to determine the viable count. The growth on HMM solid medium was calculated as the total viable yeast cells per HMM plate. The images of *Histoplasma* yeast growth on HMM plates were captured by the ChemiDoc MP Imaging System (Bio-Rad Laboratories).

### Antifungal susceptibility test

*Histoplasma*’s susceptibility to itraconazole, amphotericin B, and caspofungin was determined by the disk diffusion assay. Approximately 2 × 10^7^ yeasts were spread onto the HMM or casamino acids (2%, wt/vol) agar plates. Paper disks (1/4 in. in diameter) were placed on top of the agar medium. Ten microliters of solution containing DMSO, itraconazole (32 µg/mL, wt/vol), amphotericin B (150 µg/mL, wt/vol), or caspofungin (6.4 mg/mL, wt/vol) were dropped onto the paper disk. The plates were incubated for 5 days at 37°C under 5% CO_2_ or ambient air. The images of *Histoplasma* growth on agar plates containing antifungals were captured by the ChemiDoc MP Imaging System (Bio-Rad Laboratories). The zone of inhibition was measured using ImageJ (48).

### Statistical analysis

All experiments were conducted with at least three biological replicates. Data were analyzed by Student’s *t*-test or Welch’s *t*-test (Prism v9.4.1; GraphPad Software) for the determination of statistically significant differences, which are indicated in graphs with asterisk symbols (*, *P* < 0.05; **, *P* < 0.01; and ***, *P* < 0.001).

## SUPPLEMENTAL MATERIAL

Supplemental material spectrum.03106-24-s0001.pdf Fig. S1 to S6.

ASM does not own the copyrights to Supplemental Material that may be linked to, or accessed through, an article. The authors have granted ASM a non-exclusive, world-wide license to publish the Supplemental Material files. Please contact the corresponding author directly for reuse.
