# Supplementary material for: Elevated carbon dioxide enhances the growth and reduces the antifungal susceptibility of Histoplasma capsulatum
Source: Microbiol Spectr. 2025 May 30;13(7):e03106-24. doi: 10.1128/spectrum.03106-24 (PMC12210854; doi:10.1128/spectrum.03106-24)
Supplement: Supplemental material — Fig. S1 to S6. [file spectrum.03106-24-s0001.pdf]

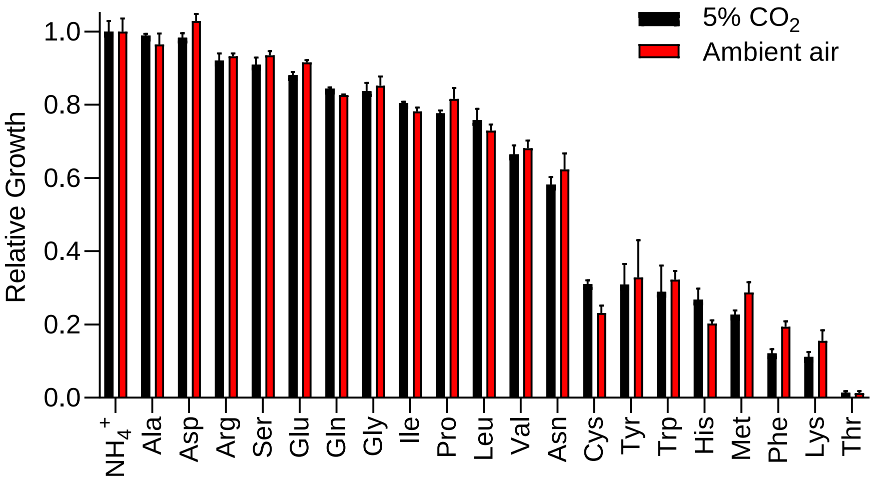

Figure S1. The growth of *Histoplasma* yeasts in individual amino acid as the sole nitrogen source. *Histoplasma* yeasts were inoculated into 3M medium at 2 × 10^6 yeasts/mL containing each individual amino acid as the sole nitrogen source. Ammonium sulfate or each individual amino acid provides 7.5 mM of nitrogen source except for cysteine (1.5 mM), tyrosine (1.25 mM), asparagine (1 mM), histidine (0.25 mM), phenylalanine (0.4 mM), and methionine (0.8 mM). Yeasts were incubated at 37°C under 5% CO_2 or ambient air (0.04% CO_2). Yeast growth was measured by determination of the optical density at 595 nm (OD595) after 7 days of incubation and normalized to the growth in ammonium sulfate. Data represent average relative growth levels ± standard deviations of results from biological replicates (n = 3).

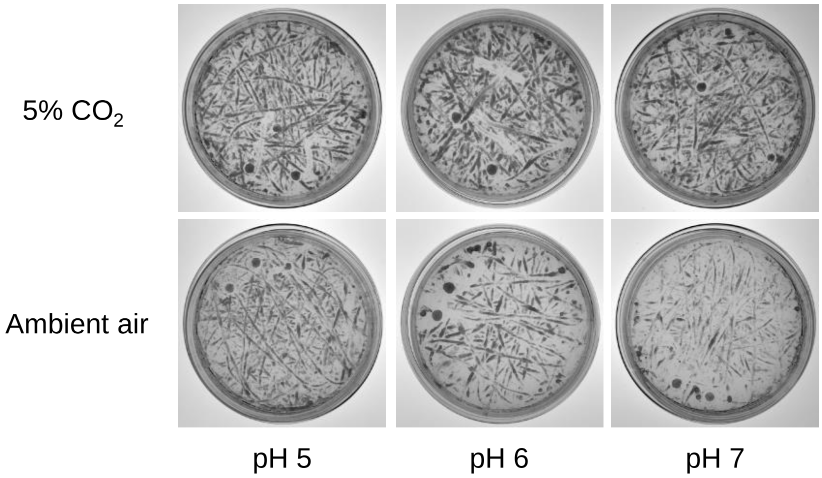

Figure S2. Elevated CO_2 enhances the growth of the Panama strain G186A on the solid medium. *Histoplasma capsulatum* G186A yeasts (5×10^6 yeasts/plate) were inoculated onto the HMM agar with pH 5, 6, and 7, respectively and incubated at 37°C under 5% CO_2 or ambient air (0.04% CO_2) for 72 h. Representative images of *Histoplasma* yeast growth (dark area) on the HMM agar with pH 5, 6, and 7, respectively under 5% CO_2 or ambient air were shown.

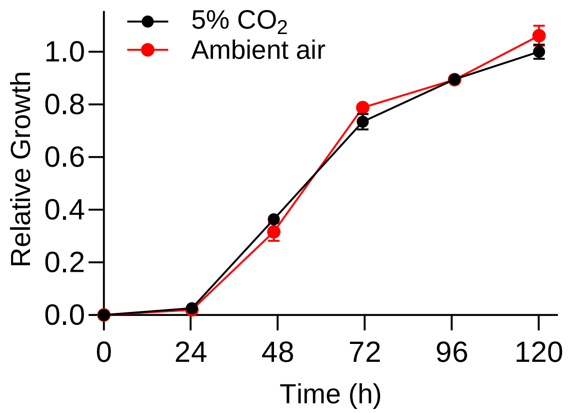

Figure S3. Elevated CO_2 does not enhance *Histoplasma*'s growth in the liquid medium. Growth curves show the growth of *Histoplasma* yeasts in the liquid HMM (pH 6.0) under 5% CO_2 or ambient air (0.04% CO_2) for 120 h. Yeast growth was measured by determination of the optical density at 595 nm (OD595) every 24 h and normalized to the maximal growth under 5% CO_2. Data represent average relative growth levels ± standard deviations of results from biological replicates (n = 3).

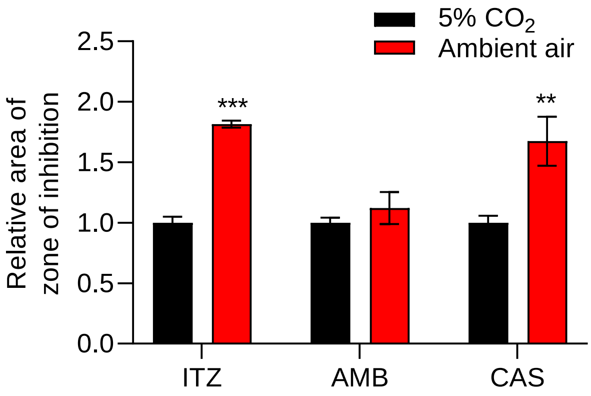

Figure S4. Elevated CO_2 reduces *Histoplasma*'s susceptibility to itraconazole and caspofungin but not amphotericin B in the amino acid medium. The antifungal susceptibility of *Histoplasma* under 5% CO_2 or ambient air (0.04% CO_2) was determined using a disk diffusion assay. Suspensions of *Histoplasma* yeasts (2×10^7 cells) were spread onto the solid 3M medium with casamino acids as the only carbon source. Disks containing ITZ (itraconazole, 32 µg/mL), AMB (amphotericin B, 150 µg/mL), and CAS (caspofungin, 6.4 mg/mL) were placed on top of the spread cells. The cells were incubated at 37°C. The area of zone of inhibition was measured after 5 days and normalized to the area of zone of inhibition under 5% CO_2. Larger zone of inhibition indicates greater antifungal susceptibility. Data represent average relative area of zone of inhibition ± standard deviations of results from biological replicates (n = 3). Asterisks indicate significant differences (**, $P < 0.01$; ***, $P < 0.001$) between 5% CO_2 and ambient air as determined by two-tailed Student's *t* test.

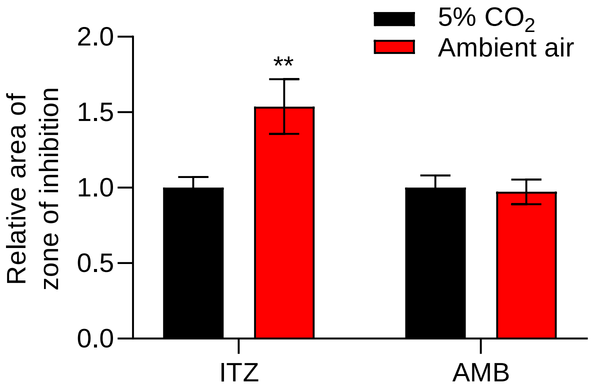

Figure S5. Elevated CO_2 reduces susceptibility to itraconazole but not amphotericin B in the Panama strain G186A. The antifungal susceptibility of *Histoplasma capsulatum* G186A under 5% CO_2 or ambient air (0.04% CO_2) was determined using a disk diffusion assay. Suspensions of *Histoplasma* yeasts (2 × 10^7 cells) were spread onto the solid HMM medium. Disks containing ITZ (itraconazole, 32 µg/mL) and AMB (amphotericin B, 150 µg/mL) were placed on top of the spread cells. The cells were incubated at 37°C. The area of zone of inhibition was measured after 5 days and normalized to the area of zone of inhibition under 5% CO_2. Larger zone of inhibition indicates greater antifungal susceptibility. Data represent average relative area of zone of inhibition ± standard deviations of results from biological replicates (n = 3). Asterisks indicate significant differences (**, $P < 0.01$) between 5% CO_2 and ambient air as determined by two-tailed Student's *t* test.

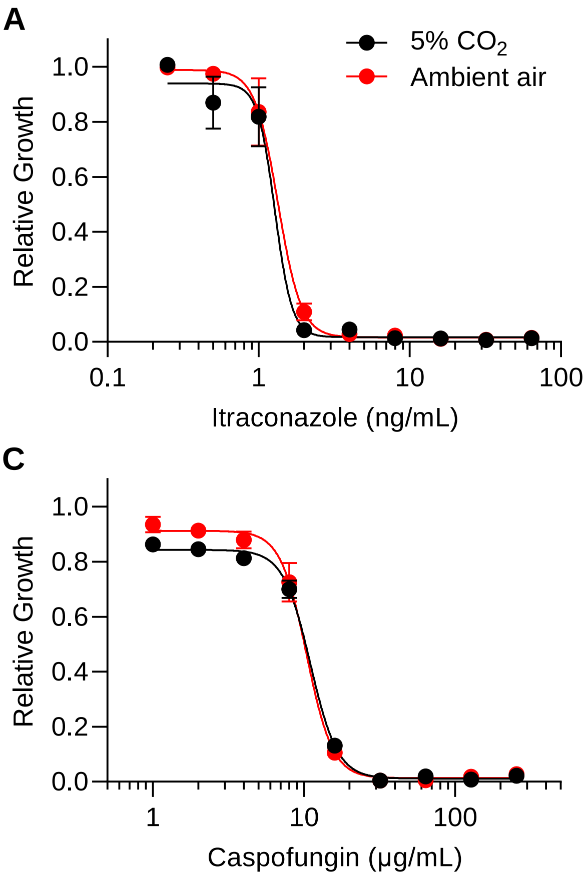

Figure S6. Elevated CO_2 does not affect *Histoplasma*'s antifungal susceptibility in the liquid media. The antifungal susceptibility of *Histoplasma* yeasts to Itraconazole **(A)**, amphotericin B **(B)**, and caspofungin **(C)** were tested using the 96-well microtiter plate microdilution assays. *Histoplasma* yeasts were inoculated into the liquid HMM medium at a density of 2 × 10^6 yeasts/mL and incubated at 37°C. Yeast growth was measured by determination of the optical density at 595 nm (OD595) after 96 h and normalized to the wells without antifungal drugs added. Data represent average relative growth levels ± standard deviations of results from biological replicates (n = 3).
